# FlhF(T368A) modulates motility in the bacteriophage carrier state of *Campylobacter jejuni*

**DOI:** 10.1111/mmi.14120

**Published:** 2018-10-23

**Authors:** Lu Liang, Ian F. Connerton

**Affiliations:** ^1^ Division of Food Sciences, School of Biosciences University of Nottingham Sutton Bonington Campus Loughborough Leicestershire LE12 5RD UK

## Abstract

The carrier state is an alternative bacteriophage life cycle by which virulent bacteriophage can persist in association with host bacteria. *Campylobacter jejuni *carrier state strains exhibit growth phase dependent motility due to a truncated flagella phenotype. Genome sequencing identified a T368A substitution in the G3 domain of the SRP‐like GTPase FlhF from *C. jejuni* PT14CP30A carrier state strains, which we hypothesized to be the cause of the complex motility phenotype. We have analyzed the role of this mutation in *C. jejuni *PT14 and demonstrated that *flhF(T368A)* leads to a large proportion of cells unable to synthesize flagella, while the remaining cells form a single flagellum at one pole leading to significantly reduced motility. The *flhF(T368A) *mutation causes a reduction in the phage adsorption constant, which leads to a decrease in infection efficiency. Down‐regulation of σ^28^ and σ^54^ dependent flagellar genes were observed as responses to the *flhF(T368A)* mutation. FlhF(T368A) protein is impaired in GTPase activity and exhibits reduced stability*. C. jejuni* carrying *flhF(T368A) *are less sensitive to bacteriophage infection and formation of the carrier state. The acquisition of *flhF(T368A) *in carrier state strains acts to prevent super‐infection and maintain association with the bacteriophage that provoked the interaction.

## Introduction

*Campylobacter jejuni *is a bacterial pathogen commonly responsible for foodborne gastroenteritis across the world. Infection can arise from a variety of food or water‐borne sources but is often associated with the consumption of contaminated poultry meat (Newell *et al*., 2011). *Campylobacter *are Gram‐negative spiral shaped bacteria that are motile by means of a single unsheathed flagellum at one or both poles of the cell (Nachamkin *et al*., 1993). During infection *Campylobacter* are frequently located within the thick mucus layer lining the intestine (Berry *et al*., 1988). Motility is clearly a significant phenotype for bacteria migrating to, and moving within the mucus to reach desired microenvironments that can facilitate growth (Lertsethtakarn *et al*., 2011). Intact flagella that support motility are essential for colonization by *Campylobacter*, and non‐motile *Campylobacter* either do not colonize the intestines of animal hosts (Mertins *et al*., 2013), or in some cases, only lead to short‐term colonization that cannot persist more than seven days postinfection (Hendrixson and DiRita, 2004).

Flagella biosynthesis is a complex process that requires the coordinate expression of over fifty genes to produce a functional flagella (Chevance and Hughes, 2008). Flagellar export in *C.* *jejuni* is controlled by a two‐component regulator FlgSR, where the FlgS histidine kinase autophosphorylates to activate the response regulator FlgR. Phosphorylated FlgR in conjunction with the σ^54^–RNA polymerase complex activates the transcription of genes encoding flagellar components, including the flagellar hook and hook‐associated proteins, and the minor flagellin FlaB. FlgSR and σ^54^ are also required for the full expression of σ^28^–dependent genes involved in flagellar biosynthesis, including the expression of *flaA* encoding the major flagellin protein (Gilbreath *et al*., 2011; Lertsethtakarn *et al*., 2011). At the front of the transcriptional cascade there are several genes transcribed from general σ^70^ promoters that are required for σ^54 ^transcription, these include *flhA*, *flhB*, *fliP*, *fliR* as well as *flhF *(Hendrixson and Dirita, 2003)*.* FlhF is reported to impart spatial and/or numerical control of flagella biosynthesis in *C.* *jejuni* (Balaban and Hendrixson, 2011), and to function at an early stage of flagella biosynthesis (Green *et al*., 2009).

Various strategies have been developed and evaluated in order to reduce the contamination of poultry by *Campylobacter* (Newell *et al*., 2011). Using bacteriophages, which are natural predators of bacteria and ubiquitous in environment, as a therapy against *Campylobacter* has demonstrated promise over the past decade (Loc Carrillo *et al*., 2005; Connerton *et al*., 2011; Kittler *et al*., 2013; Hammerl *et al*., 2014). Phage infection is initiated by phage attaching to a specific receptor followed by injection of its genome into the host cell (Choi *et al*., 2013). Bacteriophage life cycles are classed as lytic or lysogenic depending on whether the injected phage nucleic acid commits to replication and release of new phage particles upon host cell lysis or can integrate into the host genome and replicate together with the host cell (Boyd and Brussow, 2002). However, alternative bacteriophage life cycles have been described, which includes the carrier state life cycle (Abedon, 2009). The carrier state life cycle describes a situation where the host bacteria and bacteriophage persist in an equilibrium, where some of the bacteria are resistant to phage infection while others are sensitive and support phage replication, with the result that bacteria and phage maintain similar population levels in serial culture (Siringan *et al*., 2014).

Carrier state *C. jejuni *PT14CP30ACS show impaired motility and the development of a growth phase dependent sub‐population in broth culture that are associated with phage resistance (Siringan *et al*., 2014). Transmission electron microscope (TEM) images show the non‐motile bacteria to have truncated flagella (Siringan *et al*., 2014). However, the reason for this defect in flagellar biosynthesis remains unknown. This study identifies a point mutation within the *flhF* gene of *C. jejuni *carrier state cultures harboring bacteriophage CP30A and subsequently examines the mechanisms by which the *flhF(T368A) *allele modulates flagella function, bacteriophage infection and affects persistence of the carrier state of *C. jejuni *PT14.

## Results

### The FlhF(T368A) mutation is a feature of carrier state PT14CP30ACS

**Figure 1:**
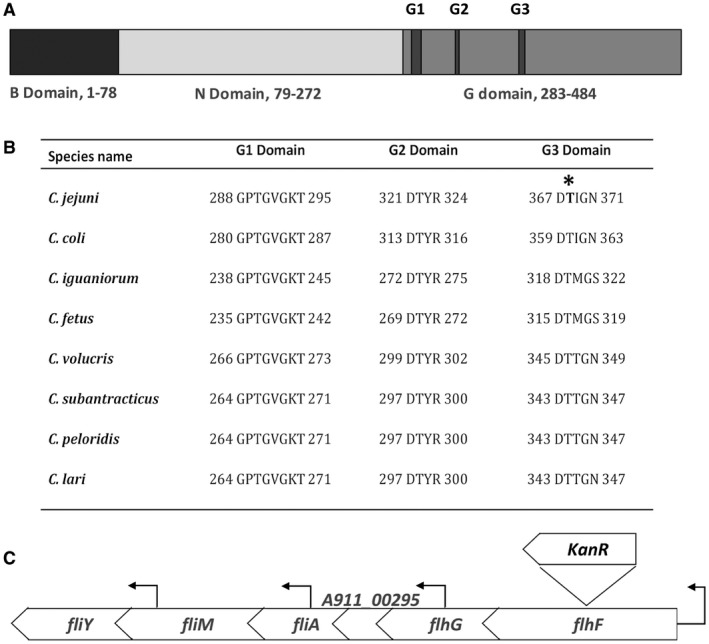
FlhF domain organisation, sequence alignment between *Campylobacter *species and *flhF* gene locus. Panel A. Domain structure of *C. jejuni *PT14 FlhF protein with reference to the amino acid residue locations: Basic domain located in the N‐terminus; N domain located in the central region; and the G domain located at the C‐terminus. The G domain functions as a GTPase and contains multiple conserved subdomains as indicated by G1, G2 and G3. Panel B. Clustal Omega alignment of the G1, G2 and G3 subdomains of the FlhF protein orthologues from *C. jejuni* *subsp. jejuni *PT14 (GenBank accession NC_018709.4), *C. coli *RM4661 (GenBank accession CP007181.1), *C. iguaniorum *1485E (GenBank accession CP009043.1), *Campylobacter fetus subsp. fetus 04/554 *(GenBank accession CP008808.1)*,* *C. volucris *LMG24379 (GenBank accession CP007774.1), *C. subantarcticus *LMG24374 (GenBank accession CP007772.1), *C. peloridis *LMG23910 (GenBank accession CP007766.1), *C. lari *RM2100 (GenBank accession CP000932.1). Panel C. The structure of the *flhF* locus of *C.* *jejuni *PT14 with the insertion site of the kanamycin resistant gene positioned in the same orientation of *flhF,* and the location of the transcription start sites marked by directional arrows above the bar (Hooton and Connerton, 2015).

Whole genome sequencing of three independent *C. jejuni *bacteriophage carrier state isolates exhibiting typical impaired motility (PT14CP30ACS‐1, PT14CP30ACS‐2 and PT14CP30ACS‐3) identified a shared adenine to guanine substitution at nucleotide 1102 within the *flhF *gene. Mapping the carrier state sequence data to the wild type *C. jejuni *PT14 genome (Brathwaite *et al*., 2013) with reference to the incumbent error profiles of the Illumina sequencing platform (Schirmer *et al*., 2016), revealed four further genes to feature G or A indels at varying frequencies within homopolymer regions that signify phase variation (Table 1). The genome sequences otherwise retained the phase variable gene profiles of the motile progenitor. The *flhF* mutation creates a T368A substitution in the FlhF protein when compared to the wild type sequence of *C. jejuni *PT14. The NCBI database currently contains 604 protein sequences of FlhF from *Campylobacter* and *Helicobacter* species, which are well conserved, and all of which feature a threonine residue at the equivalent sequence position. The substitution is therefore unique and has the potential to modify the function of FlhF (Fig. 1).

**Table 1:** Nucleotide changes present in the genome sequences of bacteriophage carrier state cultures.

Accession number	Gene product	Changes in coding region	Frequency	Reading frame change	Homopolymer change	Occurence in CSLC cultures
A911_00305	FlhF GTPase	A to G substitution pos.1102	100%	T368A	–	1,2,3
A911_05520	1,3‐galactosyltransferase	G deletion pos.341	28–33%	G114 fs	11G→10G	1,2,3
A911_06880	KpsC capsular polysaccharide exporter	A deletion pos.439	48–56%	R147 fs	6A→5A	1,2,3
A911_06906	SAM dependent methyltransferase	G deletion pos.402	84–88%	Y135 fs	9G→8G	1,2,3
A911_07000	alpha‐2,3‐sialyltransferase	A insertion pos.544	16–18%	I182 fs	8A→9A	1,3
A911_08080	Lipoprotein	G deletion pos.506	61–69%	G169 fs	10G→9G	1,2,3

PT14CP30ACS‐1, PT14CP30ACS‐2 and PT14CP30ACS‐3 relative to the reference sequence of *C. jejuni* PT14 (CP003871). fs indicates a frameshift (phase‐off).

FlhF together with the signal sequence‐binding protein Ffh and signal recognition particle (SRP) receptor FtsY, form a unique subfamily within the SIMIBI‐type nucleoside triphosphate‐binding protein class referred to as SRP GTPase proteins, which are universally conserved (Leipe *et al*., 2002; Zanen *et al*., 2004). Unlike Ffh and FtsY that function as a SRP‐SRP Receptor (SRP‐SR) heterodimer in the presence of GTP, FlhF proteins form a stable homodimer structure through interaction of their GTP binding domains (Bange *et al*., 2007). The GTP binding domain (G domain) is located toward the C‐terminus of FlhF, which shares conserved amino acid residues with the G domains of Ffh and FtsY. FlhF also contains conserved B and N domains (Fig. 1A). The B domain functions to regulate dimerization in the presence of GTP (Bange *et al*., 2007), while the N domain is thought to stabilize the GTP bound state by rearranging the SRP conformation upon ribosome binding, and further prime it for the formation of subsequent homodimer or heterodimer complexes (Halic *et al*., 2006). The G domain defines the GTP binding site, within which are five conserved nucleotide‐binding elements (G1‐G5) (Eichler and Moll, 2001). The functions of the SRP GTPases include translation, protein translocation, signal transduction, regulation of cell polarity and possibly cell division (Bulyha *et al*., 2011). The observed T368A mutation is identified in the third GTP binding element of the G domain within FlhF (G3, Fig. 1B).

### FlhF is required for motility of C. jejuni PT14 but FlhF(T368A) has a negative impact on motility

In order to determine the effect of *flhF(T368A) *on the motility of *C. jejuni* independent of phage replication or phase variable changes in the carrier state, three *flhF *constructs were prepared in *C. jejuni *PT14. *C. jejuni *PT14*flhF::kan* was created by inserting a kanamycin resistance gene in the same orientation to inactivate *flhF *(Fig. 1C)*. *Complements of the *flhF::kan* mutant with either *flhF* wild type or *flhF(T368A) *alleles were prepared by directed insertion into the *A911_00230 *pseudogene*.* As the *flhF *gene sequence is identical between *C. jejuni *PT14 and *C. jejuni *NCTC11168, similar constructs were prepared in *C. jejuni *NCTC11168 in parallel experiments.

**Figure 2:**
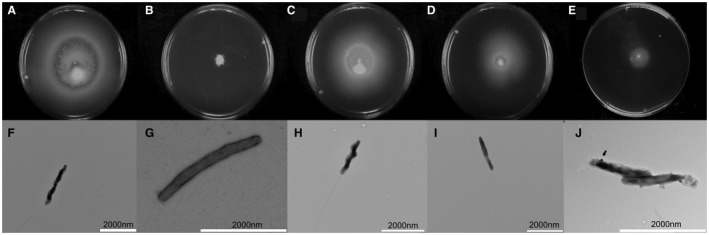
Swarming motility assays and TEM images. Panels A to E show swarming growth after 48 h from center inoculums of 0.4% of agar (w/v) MH plates, where the diameters of the bacterial halos formed are recorded in the panel key below. Panels F to J show TEMs of uranyl acetate stained bacteria and the flagellated bacteria population were determined (*n* = 100). Panels A and F are wild type *C. jejuni *PT14 (*d* = 77 mm); B and G *C. jejuni PT14flhF::kan *(*d* = 6 mm)*;* C and H *C. jejuni *PT14*flhF::kan 00230::flhF‐cat *(*d* = 68 mm)*; *D and I *C. jejuni *PT14*flhF::kan 00230::flhF(T368A)‐cat *(*d* = 39 mm)*; *E and J *C. jejuni *PT14CP30ACS (*d* = 17 mm). The arrowhead in panel J indicates a bacteriophage binding to the surface of a *C. jejuni *PT14CP30ACS cell.

The swarming motility of wild type *C. jejuni *PT14 and its *flhF *mutants were assessed on 0.4% w/v agar plates. As expected, the wild type *C. jejuni *strain was fully motile (Fig. 2A) with the cells exhibiting typical bipolar flagella when observed using TEM (Fig. 2F). Inactivation of the *flhF* gene resulted in a non‐motile phenotype (PT14*flhF::kan*) where flagella structures were absent from all the cells imaged by TEM (Fig. 2B and G). The *flhF* mutant cells also appeared curved rather than the spiral form of the wild type that typifies campylobacters. Morphometric measurements indicate the reduced curvature is associated with a significant increase in the helical pitch of the cells compared to wild type (Table 2). Complementation of the *flhF* mutation in *C. jejuni *PT14 (*flhF::kan 00230::flhF‐cat)* restored 90% motility compared to wild type *C. jejuni *PT14 with 88% of cells exhibiting polar flagella structures by TEM (Fig. 2C and H). These cells had also notably regained spiral morphology. Alternatively, complementation with *flhF(T368A)* produced strains (*flhF::kan 00230::flhF(T368A)‐cat*) with approximately 50% of the swarming motility of the wild type with TEM showing 24% of the cells to have single polar flagella structures (Fig. 2D and I) or none (Supplementary File 1D). The mean contour lengths of the flagella of these strains were also significantly reduced compared to the wild type and the wild type complement strain, as determined from TEM images (Table 2). Motility in *C. jejuni* shows temperature dependence, with growth temperature of 42°C favoring motility with longer flagella compared to lower growth temperatures (Wösten *et al*., 2010). The swarming motility of the wild type, *flhF* mutant and complement strains were assessed at 30, 37 and 42°C. Wild type and wild type complement strains showed the greatest motility at 42°C, reduced motility at 37°C and no motility at 30°C. Whereas the *flhF(T368A)* complement stain was only measurably motile at 42°C (Table 2).

**Table 2:** Summary of motility characteristics of *flhF* mutant, complement and carrier state strains.

*C. jejuni* strain	Cell shape	Cell pitch (µm)	Flagellated cells (%)	Swarming motility (mm)^a^			Flagellar length (µm)
				42^o^C	37^o^C	30^o^C	
Wild type PT14	Spiral	0.91 ± 0.16	94 ± 3	75 ±7	23 ± 3	≤3	4.74 0.73
*flhF::kan*	Curved	2.36 ± 0.33^b^	0	≤3	≤3	≤3	0
*flhF::kan,00230::flhF‐cat*	Spiral	0.91 ± 0.12	88 ± 4	68 ± 4	17 ± 2	≤3	4.51 ± 1.3
*flhF::kan,00230::flhF(T368A)‐cat*	Curved	1.90 ± 0.37^b^	24 ± 3	38 ± 6	≤3	≤3	3.16 ± 0.91^c^
PT14CP30ACS‐1	Curved	1.67 ± 0.47^b^	5 ± 3	14 ± 2	≤3	≤3	0.69 ± 0.14^b^^,^ c

Means ± SD;

a Growth diameters on 48 h MH motility plates

b indicates *p* < 0.05 relative to wild type

c measurements from the minority of cells with flagella.

By comparison, the carrier state strain *C. jejuni *PT14CP30ACS was non‐motile under these conditions (Fig. 2E). TEM revealed 95% of the cells to have no flagella (Fig. 2J), with the remaining cells exhibiting either short or aberrant flagella positioned at non‐polar locations (Supplementary File 1A and B). Phage CP30A was also often observed to be adsorbed to the surface of the cells as indicated by the arrow in Fig. 2J and occasionally encapsulated (Supplementary File 1C).

The swarming motility of *C. jejuni *NCTC11168 and its *flhF *mutants were also determined. Similar to *C. jejuni *PT14, wild type *C. jejuni *NCTC11168 was motile while the *flhF::kan *derivative was non‐motile. Complementation the *flhF::kan *mutant with either *flhF *or *flhF(T368A)* successfully restored its motility but as observed for *C. jejuni *PT14, wild type *flhF *complementation produced a greater recovery in motility than *flhF(T368A)* complementation (data not shown).

### FlhF is required for full expression of flagellar associated σ^28 ^and σ^54^ dependent genes but FlhF(T368A) is impaired

**Figure 3:**
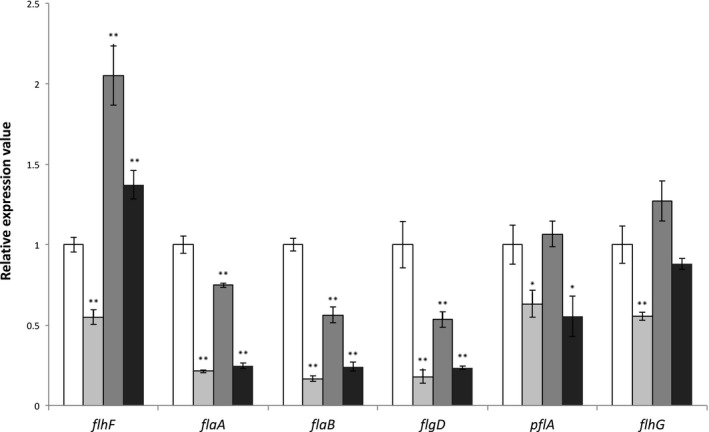
Transcript levels of flagellar associated genes. Relative transcription levels of *flhF*, *flaA, flaB, flgD pflA *and *flhG* were determined by qPCR. Values represent the means of RNA populations extracted from triplicate cultures. The housekeeping gene encoding phosphoglycerokinase (*pgk*) was used to normalize the data. *C. jejuni *PT14 wild type (□); *C. jejuni *PT14*flhF::kan *(■)*;* *C. jejuni *PT14 *flhF::kan 00230::flhF‐cat *(■)*;* *C. jejuni *PT14*flhF::kan 00230::flhF(T368A)‐cat *(■). * indicates gene expression value of *flhF *derivative is significantly different to that of wild type *p* < 0.05; ** indicates *p* < 0.01.

Insertion of the kanamycin gene within *flhF* resulted in 2‐fold lower expression from the native promoter of *flhF* compared to wild type *C. jejuni *PT14 as measured by qRT‐PCR (*p* < 0.01; Fig. 3). Ectopic complementation of *flhF *(*flhF::kan 00230::flhF‐cat)* restored *flhF *transcription to produce levels 2‐fold greater than wild type *C. jejuni *PT14 (*p* < 0.01). The second gene of the *flhF* operon is *flhG*, which is reported to encode an ATPase that represents a member of the ParA superfamily of proteins that regulate cell division, and in the case of *C. jejuni* is reported to mediate the site and numerical control of flagellar synthesis (Gulbronson *et al*., 2016). Inactivation of *flhF* similarly reduced the transcription of the *flhG* gene relative to wild type (*p* < 0.01), and transformant cells carrying ectopic copies of *flhF* also restored *flhG* transcription to wild type levels. The increase in transcription of *flhG* as a consequence of second site *flhF* expression would mitigate against any effect of the kanamycin gene insertion. Ectopic complementation with *flhF(T368A)* (*flhF::kan 00230::flhF(T368A)‐cat*) also rescued transcription of *flhF *and *flhG* but the levels were significantly lower than for the wild type complement (*p* < 0.01).

We next determined if *flhF* was essential for the expression of σ^28^ and σ^54^ –dependent flagellar associated genes, and whether the expression of *flhF(T368A) *affected the regulation of these genes (Fig. 3). The *flaA* gene encoding the major flagellin FlaA was down‐regulated 4.7‐fold in the *flhF* mutant (*p* < 0.01). Ectopic expression of *flhF* restored *flaA* expression to 75% of wild type, whereas ectopic expression of *flhF(T368A)* did not produce a significant increase in *flaA* expression over the *flhF* mutant. In contrast to *flaA* that has a σ^28^‐dependent promoter, the minor flagellin encoding gene, *flaB*, has a σ^54^‐dependent promoter (Guerry *et al*., 1991; Wassenaar *et al*., 1993). The *flaB* gene was down‐regulated 6‐fold in the *flhF* mutant (*p* < 0.01), and ectopic expression of *flhF* also increased *flaB* expression over the *flhF* mutant but this increase was not evident upon ectopic expression of the *flhF(T368A)* allele (*p *> 0.05). Similarly, *flgD* (encoding a hook assembly protein) uses a σ^54^‐dependent promoter (Balaban *et al*., 2009) that is reduced in the *flhF* mutant and increases upon ectopic expression of wild type *flhF* but not *flhF(T368A)*. Expression of the *pflA* gene encoding the paralyzed flagella protein was also significantly reduced in the *flhF* mutant (*p *< 0.05) and recovered upon expression of wild type *flhF* but not *flhF(T368A)*. In the absence of *pflA* expression cells produce intact flagella structures but are non‐motile (Gao *et al*., 2014). In summary, ectopic complementation of inactivated *flhF* mutants of *C. jejuni *PT14 (*flhF::kan 00230::flhF‐cat*) resulted in significant increases in the expression of σ^28^ and σ^54^ regulated genes (*p* < 0.05), but these increases were not evident upon expression of *flhF(T368A)*.

### C. jejuni PT14 flhF mutants are less sensitive to phage infection

*Campylobacter*‐specific bacteriophages of the *Myoviridae* subfamily *Eucampyvirinae* (Javed *et al*. 2014) were applied to bacterial lawns formed from either *C. jejuni *PT14, or PT14*flhF::kan* or PT14*flhF*::*kan* 00230::*flhF‐cat* or PT14*flhF*::*kan* 00230::*flhF(T368A)‐cat* at a test dilution of log_10_ 7 pfu ml^–1^ in order to investigate the impact of the *flhF* related genotypes on their ability to act as a host for bacteriophage infection. Data are presented as the efficiency of plating (EOP) for each phage relative to the titer on wild type *C. jejuni* PT14 for each of the host strains in Table 3. Phages CP220, ɸ3 and ɸ15 are classified as group II (Cp220likevirus) based on their genome sizes (180 and 190 kb) and head diameters (Sails *et al*., 1998), and the remaining phages are group III (Cp8unalikevirus) with genome sizes of approximately 140 kb (Connerton *et al*., 2004; Siringan *et al*., 2011; Firlieyanti *et al*., 2016). Group II phages are flagellotropic, which require a functional flagellar to infect (Coward *et al*., 2006; Scott *et al*., 2007a; Baldvinsson *et al*., 2014; Lis and Connerton, 2016), whereas group III phages can be affected by loss of motility they show dependence on capsular polysaccharide structures (Sørenson *et al*., 2011; Lis and Connerton, 2016). The *flhF* inactive strain showed reduced EOP for all group III phages compared to wild type (*p* < 0.05), and no replication of the group II phages (Table 3). Complementation of the *flhF *mutant with wild type *flhF* restored sensitivity to all the phages tested, however, the EOPs of CP220, Ø3 and CLP6 remained significantly reduced compared to wild type *C. jejuni* PT14. The *flhF(T368A) *complement strain exhibited significantly reduced EOPs for the group III phages similar to that observed for the knock‐out *flhF *mutant, and similarly no plaques were formed for the group II phages on *flhF(T368A)* (Table 3). Group III phage retained the ability to replicate on the *C. jejuni *PT14 *flhF *mutant and have been demonstrated to enter the carrier state (Siringan *et al*., 2014). Group III phage CP30A were therefore selected to investigate the replication parameters of the *flhF *derivatives. The adsorption constants (*k*) for phage CP30A binding to *C. jejuni *PT14 *flhF *mutant or the *flhF(T368A) *complement show a 1.7‐fold decrease compared to wild type (*p* < 0.05; Table 4), whereas the wild type complement of *flhF *showed no significant difference (*p* > 0.05). The burst size revealed no difference between *C. jejuni *PT14 (1.95 ± 0.54 pfu cell^–1^) and its *flhF *mutant derivatives. The latent period for all the *C. jejuni *strains tested was approximately 60 min.

**Table 3:** Efficiency of plating of bacteriophages replicating on *flhF* mutant and derivatives compared to *C. jejuni* PT14.

*C. jejuni *strains	Group II phage			Group III phage				
	CP220	Ø3	Ø15	CP30A	CP8	CPX	CLP6	CLP47
PT14	1 ± 0.10	1 ± 0.13	1 ± 0.33	1 ± 0.12	1 ± 0.13	1 ± 0.07	1 ± 0.13	1 ± 0.06
*flhF::kan*	ND	ND	ND	0.57 ± 0.03^a^	0.50 ± 0.22^a^	0.40 ± 0.18^a^	0.26 ± 0.09^a^	0.17 ± 0.04^a^
*flhF::kan,00230::flhF‐cat*	0.06 ± 0.02^a^	0.33 ± 0.07^a^	0.72 ± 0.12	0.74 ± 0.13	0.88 ± 0.22	0.76 ± 0.25	0.63 ± 0.05^a^	0.88 ± 0.17
*flhF::kan,00230::flhF(T368A)‐cat*	ND	ND	ND	0.51 ± 0.06^a^	0.63 ± 0.13^a^	0.44 ± 0.07^a^	0.57 ± 0.09^a^	0.35 ± 0.10^a^

ND indicates none detected;

a indicates EOP in the test group is significantly different to the EOP of the control wild type *C. jejuni* PT14 group (*p* < 0.05).

**Table 4:** Replication parameters for phage CP30A on different host strains.

*C. jejuni *host strain	Adsorption constant (k) × 10^–10^ (ml min^–1^)	Burst size (pfu cell^–1^)	Latent period (min)
PT14	1.13 ± 0.42	1.95 ± 0.54	60
PT14*flhF::kan*	0.65 ± 0.02^a^	1.91 ± 0.69	60
PT14*flhF::kan 00230::flhF‐cat*	1.19 ± 0.24	1.91 ± 0.38	60
PT14*flhF::kan 00230::flhF(T368A)‐cat*	0.63 ± 0.24^a^	1.91 ± 0.87	60

a indicates *p *< 0.05 relative to wild type.

**Figure 4:**
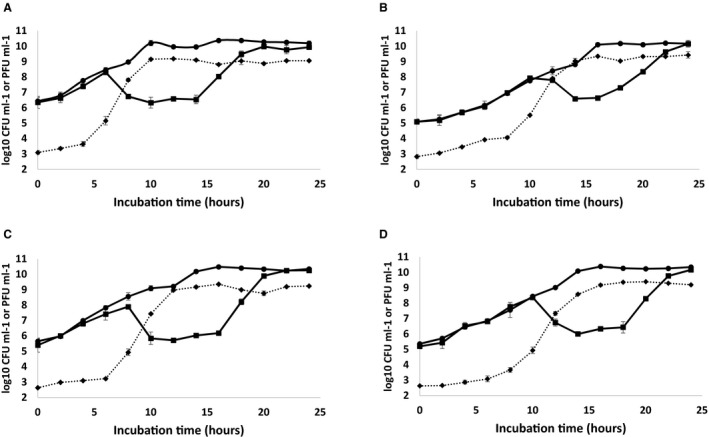
Growth characteristics of *C. jejuni* PT14 wild type and its *flhF *mutants upon infection by bacteriophage CP30A. Broth cultures containing 4 log_10_ CFU *C. jejuni* were pre‐incubated for 2 h before infection with 2 log_10_ PFU bacteriophage CP30A (time zero). Sample aliquots were removed every 2 h to determine the bacterial counts and phage titers. Panels A. *C. jejuni* PT14, B. *C. jejuni* PT14*flhF::kan*, C. *C. jejuni *PT14*flhF::kan 00230::flhF‐cat*, D. *C. jejuni* PT14*flhF::kan 00230:: flhF(T368A)‐cat*. The panels show the viable counts for a parallel uninfected control culture (●); viable counts for the bacteriophage CP30A infected *C. jejuni* cultures (■); bacteriophage CP30A titers from the infected cultures (◆).

We further investigated the growth of host cultures in response to phage replication. Phage CP30A was used to infect *C. jejuni *PT14, PT14*flhF::kan*, PT14*flhF::kan 00230::flhF‐cat* and PT14*flhF::kan 00230::flhF(T368A)‐cat* in broth cultures. Increases in phage titer were observed in all cases when the density of the bacteria was > 7 log_10_ CFU ml^–1^ – the phage proliferation threshold (Cairns *et al*., 2009). In the wild type *C. jejuni* PT14 cultures, an increase in phage titer was accompanied by a 2.2 log_10_ CFU ml^–1^ fall in the bacterial count at 8 h, whereafter the counts remained static for 6 h before recommencing growth after 16 h (Fig. 4A). Phage replication was impaired in the *flhF* knock‐out mutant and the *flhF(T368A)* complement as indicated by delays of 2–4 h in the bacterial population crash and concomitant rise in phage titer. As observed previously for laboratory cultures, bacterial growth recommenced after the crash with the development of phage resistance (Scott *et al*., 2007b). However, the static period following the phage‐induced population crash of the *flhF* mutant was reduced to 4 h followed by a comparatively reduced rate of recovery (Fig. 4B). The static period was reinstated in the complement strain PT14*flhF::kan 00230::flhF‐cat* and to a lesser extent for PT14*flhF::kan 00230::flhF(T368A)‐cat* (Fig. 4C and D), suggesting more efficient reinfection of the *C. jejuni *with flagellar structures.

Individual colonies were recovered from the enumeration plates of the cultures post static phase (*n* = 100) and their sensitivity to infection by group II (CP220, Φ3, Φ15) and III (CP30A, CPX, CLP6 and CLP47) phages compared to non‐infected cultures. Of these, 10% of the isolates recovered post phage CP30A infection of *C. jejuni *PT14 wild type and 7% for the *flhF* complement (PT14*flhF::kan 00230::flhF‐cat*) had entered carrier state life cycle with continued CP30A phage association and impaired motility. The remaining isolates retained their susceptibility to phage CP220, Φ3, Φ15, CP30A and CPX. However, 53% of the colonies recovered post CP30A infection of *C. jejuni *PT14 wild type had developed resistance to phages CLP6 and CLP47, which distinguished them from other group III phage. Wild type complement (PT14*flhF::kan 00230::flhF‐cat*) isolates did not develop resistance to phage CLP6 and CLP47. No carrier state isolates emerged from the *flhF* mutant (PT14*flhF::kan*) or the *flhF(T368A)* complement (PT14*flhF::kan 00230::flhF(T368A)‐cat*). The frequency of infection of these strains is reduced, which is also likely to effect the formation of the carrier state. However, isolates from these cultures had acquired resistance to phages CLP6 and CLP47, again marking the phages CLP6 and CLP47 as different to the group III phages CP30A and CPX.

To assess if the carrier state strains emerging from CP30A infection of wild type cultures had acquired the *flhF(T368)* mutation, the *flhF *gene was PCR amplified from genomic DNAs prepared from plate swabs of carrier state cultures and the amplicons sequenced with reference to the presence of the base calls for the wild type and *flhF(T368)* alleles. The *flhF(T368A)* allele appeared as the dominant *flhF* genotype in these cultures but the wild type allele was also evident at frequencies ≤ 10^–3^. The presence of a wild type subpopulation would provide a mechanism by which phage titers are maintained through bacteriophage replication in a subpopulation of cells, and an explanation for the emergence of motile types observed in synchronous broth cultures (Siringan *et al*., 2014).

### FlhF(T368A) shows reduced thermal stability and GTPase activity

**Figure 5:**
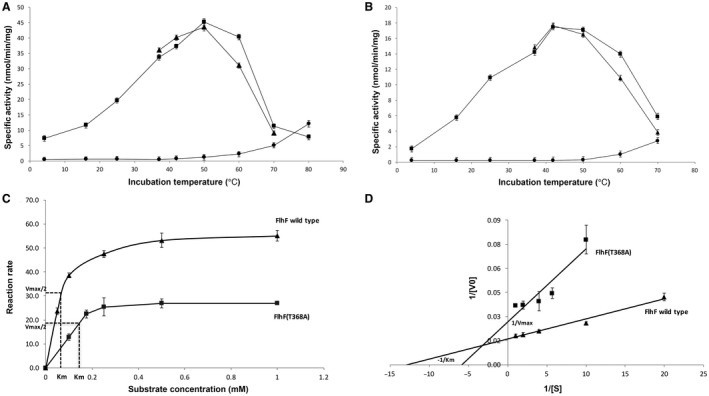
GTPase activities of purified FlhF and FlhF(T368A) proteins. Panels A and B show temperature profiles of the GTPase activities for FlhF proteins: Panel A. FlhF wild type protein; Panel B. FlhF(T368A) protein, where (■) denotes the FlhF GTPase activity temperature profile; (▲) denotes FlhF protein preheated at the recorded temperature for 5 min before commencing the reaction; (●) represents the GTP self‐hydrolysis control. Panels C (Michaelis‐Menten plots) and D (linear regression Lineweaver‐Burk plots) show kinetic plots for GTPase activities of FlhF (▲) and FlhF(T368A) (■) proteins.

FlhF protein is reported to have GTPase activity that is required for the correct biosynthesis of functional flagella (Balaban *et al*., 2009). Wild type FlhF and FlhF(T368A) proteins were expressed as N‐terminal His_6_‐tagged fusions in *Escherichia coli* using a pET28b vector. For wild type FlhF, *E. coli* BL21DE3 Star cells were used for protein expression, but for FlhF(T368A) it was necessary to use Origami 2 DE3 pLysS cells to overcome inherent protein instability and prevent inclusion body formation. The proteins were purified by a combination of Ni‐affinity and Superose 12 size‐exclusion chromatography. GTPase activities were measured by detecting the release of free phosphate (*P*_i_) as a product of GTP hydrolysis. The temperature optimum of FlhF GTPase was 50°C with a specific activity of 45 nmol min^–1^ mg^–1^ compared to a temperature of optimum of 42°C and a specific activity of 17.5 nmol min^–1^ mg^–1^ for FlhF(T368A) (Fig. 5A and B respectively). The specific GTPase specific activity of wild type FlhF was 2‐fold greater than that of FlhF(T368A) at 42°C that represents the growth temperature that *C. jejuni* exhibits the greatest motility. Consistent with the relative thermal sensitivity of the FlhF proteins, the wild type protein produced a 7 ± 0.03% increase in GTPase activity upon pre‐incubation at 42°C for 5 min that was not observed with FlhF(T368A). However, pre‐incubation with 0.2 mM GTP at 42°C for 5 min with correction for substrate conversion at time zero, produced a 12 ± 0.1% increase in the GTPase activity for wild type FlhF compared to a 14 ± 0.5% increase for FlhF(T368A). The enzymes show substrate stabilization but FlhF(T368A) shows a significantly greater response.

Kinetic parameters at the optimum growth temperature of *C. jejuni *of 42°C were determined for both FlhF and FlhF(T368A) (Fig. 5C and D). The *K*_M_ for FlhF was 0.075 mM and the *V*_max_ 62.5 nmol min^–1^ mg^–1^, and by comparison the GTPase activity of FlhF(T368A) was impaired with a *K*_M_ of 0.1736 mM and *V*_max_ 37.74 nmol min^–1^ mg^–1^. The *k*_cat _for FlhF and FlhF(T368A) were calculated as 0.0115 s^–1^ and 0.007 s^–1^ respectively.

## Discussion

The complex motility phenotypes of bacteriophage carrier state *C. jejuni* prompted this study (Siringan *et al*., 2014). Motility is essential for *C. jejuni* to colonize animal hosts and cause disease (Lertsethtakarn *et al*., 2011), and accordingly carrier state strains show reduced adhesion and invasion of human colonic epithelial cells (Brathwaite *et al*., 2015) and fail to colonize chickens (Siringan *et al*., 2014). *C. jejuni* possess a characteristic high‐torque flagellar motor that enables swimming at viscosities that effectively immobilize other enteric bacteria, and has been proposed to represent a disproportional leap in the evolution of the flagellar motor structure (Chaban *et al*., 2018). The flagellar patternation of *C. jejuni* is characterized as amphitrichous with a single flagellum located at each of the cell poles. *Campylobacter* flagella biosynthesis is complex but control of the number of flagellar and their polar location are dependent upon the proper expression of the *flhFflhG* operon (Balaban and Hendrixson, 2011). We have identified a point mutation in the *flhF* gene of carrier state *C. jejuni* strains in association with bacteriophage CP30A that we hypothesized was responsible for the observed impaired motility. The mutation represents a unique substitution of T368A within the third GTP binding element of the conserved G domains of FlhF (Bange *et al*., 2007).

As reported previously inactivation of *flhF* produces a non‐motile phenotype in *C. jejuni* (Balaban *et al*., 2009). Ectopic complementation of the *flhF *mutation at the *00230 *pseudogene locus of *C. jejuni* PT14 restored motility. However, complementation with *flhF(T368A)* led to reduced motility compared to wild type and the wild type complement, indicating that the *flhF(T368A)* mutation present in CP30A associated carrier state strains will confer an impaired motility phenotype independent of the phage association/replication or the presence of phase variable reading frames. Transmission electron microscopy of *C. jejuni* PT14 and the *flhF* mutant derivatives confirmed *C. jejuni *PT14 to have a typical bi‐polar flagella and that the *flhF::kan* inactivated mutant was devoid of flagella structures. Wild type complement cells (PT14*flhF::kan 00230::flhF‐cat*) regained a polar flagella, whereas the cells expressing the *flhF(T368A)* allele (PT14*flhF::kan00230::flhF(T368A)‐cat*) showed single flagella structures in 24% of the cells examined and the rest none. These data confirm the critical importance of the GTPase component of FlhF in *C. jejuni* (Balaban *et al*., 2009).

Insertional inactivation of *flhF* significantly reduced the expression of the *flhF* and the downstream gene *flhG* (Fig. 1C). The transcript levels of *flhF* and *flhG* could be restored to wild type or greater levels by ectopic expression of *flhF* under the native σ^70^ promoter. The wild type *flhF* complement achieves double the relative expression of wild type implying the two *flhF* promoters present are functioning similarly to additively increase the transcript level but this does not extend to *flhG*, which remains dependent on the resident *flhF* promoter. Ectopic expression of the *flhF(T368A)* allele also increased *flhF *and *flhG *transcription levels over the *flhF* inactivated mutant but were significantly less than the wild type *flhF* complement. These data support the contention that the *flhF* gene product exerts a positive feedback on *flhF *and *flhG* expression. This could be accomplished either by direct or indirect activation of the *flhF flhG* promoter, or alternatively via specific stabilization of the *flhF flhG* mRNA.

To gain a greater understanding of the role of *flhF(T368A) *on flagellar biosynthesis we examined the expression of σ^28^ (the flagellar sigma factor regulating the major flagellin encoding gene *flaA*) and σ^54 ^(sigma factor regulating genes encoding flagellar basal body, hook, the minor flagellin *flaB* and anti‐sigma factor *flgM*) genes in the mutant and wild type cultures. In the absence of *flhF, *the expression levels of *flaA *(σ^28 ^transcription‐dependent), *flaB* and *flgD* (σ^54 ^transcription‐dependent) were all significantly reduced, and less severe but still significant downregulation was observed for *pflA*. Hendrixson and DiRita (2004) hypothesized that *flhF *exerted it’s influence at the start of the σ^54 ^transcriptional cascade, and therefore deletion of *flhF *would result in improper transcription of the downstream genes and reduced levels of σ^54^ dependent gene expression, which would account for the observations in this study that *flaB *and *flgD *transcription becomes downregulated. However, Correa *et al*. (2005) reported that in *Vibrio cholera*e, *flhF *is also essential for the expression of several σ^28 ^dependent flagellar genes including the major flagellin homologue. In contrast Hendrixson and DiRita (2003) found that in *C. jejuni* 81–176, σ^28 ^dependent *flaA *was largely unaffected by deletion of *flhF*. Subsequently Balaban *et al*., (2009) reported that a *flhF* deletion mutant of *C. jejuni* 81–176 expressed 50% less *flaA* but point mutations located in the G1 and G2 domains of *flhF* increased *flaA* expression. More recently Ren *et al*. (2018) have reported 5.3‐fold down regulation of the *flaA* gene in a *flhF* insertional inactivation mutant constructed in *C. jejuni* NCTC11168 (as we also observed). In this study, disruption of *flhF *led to a significant reduction in the expression of *flaA*. Ectopic expression of wild type *flhF *in the *flhF* mutant led to increases in the expression of σ^28 ^and σ^54^ dependent genes that was not observed upon expression of the *flhF(T368A) *allele. Instead, the *flaA, flaB *and *flgD* transcript levels were not significantly different to those recorded for the *flhF::kan *mutant. The mechanism by which FlhF enables the transcription of the flagellar associated genes appears dependent on assembling the FlhF GTPase domain since the *flaA, flaB *and *flgD* transcript levels fail to recover upon expression of FlhF(T368A) exhibiting impaired GTPase. We have noted instability in the FlhF(T368A) protein that may hinder recruitment of components required to affect the expression of downstream flagellar components, which may also provide an explanation as to the differential effects of mutants affected in FlhF GTPase activity (Balaban *et al*., 2009), if the mutants also alter FlhF structural dependencies. The expression of the σ^28 ^and σ^54 ^dependent genes determined in this study for the *flhF(T368A) *complement relative to wild type *C. jejuni *PT14 are consistent with the transcriptome (RNA‐seq) study reported for a *C. jejuni *PT14CP30A carrier state culture (Brathwaite *et al*., 2015). The exception to this is the expression of *flaB, *which is down regulated in the *flhF(T368A) *complement strain relative to wild type *C. jejuni *PT14, as compared to a 3‐fold up regulation reported for the *C. jejuni *PT14CP30A carrier state strain. Phage transcription indicative of the progression toward replication was evident in the carrier state cultures (0.2% of the total reads), and where the abundance of the major capsid protein transcripts represented > 95% of all the normalized read counts (Brathwaite *et al*., 2015). We therefore posit that the up regulation of *flaB *in the carrier state *C. jejuni *is a consequence phage transcription/replication. In this context FlaB has recently been reported to confer defensive properties against phage infection, which affects regrowth of the host following lysis of laboratory cultures (Lis and Connerton, 2016).

**Figure 6:**
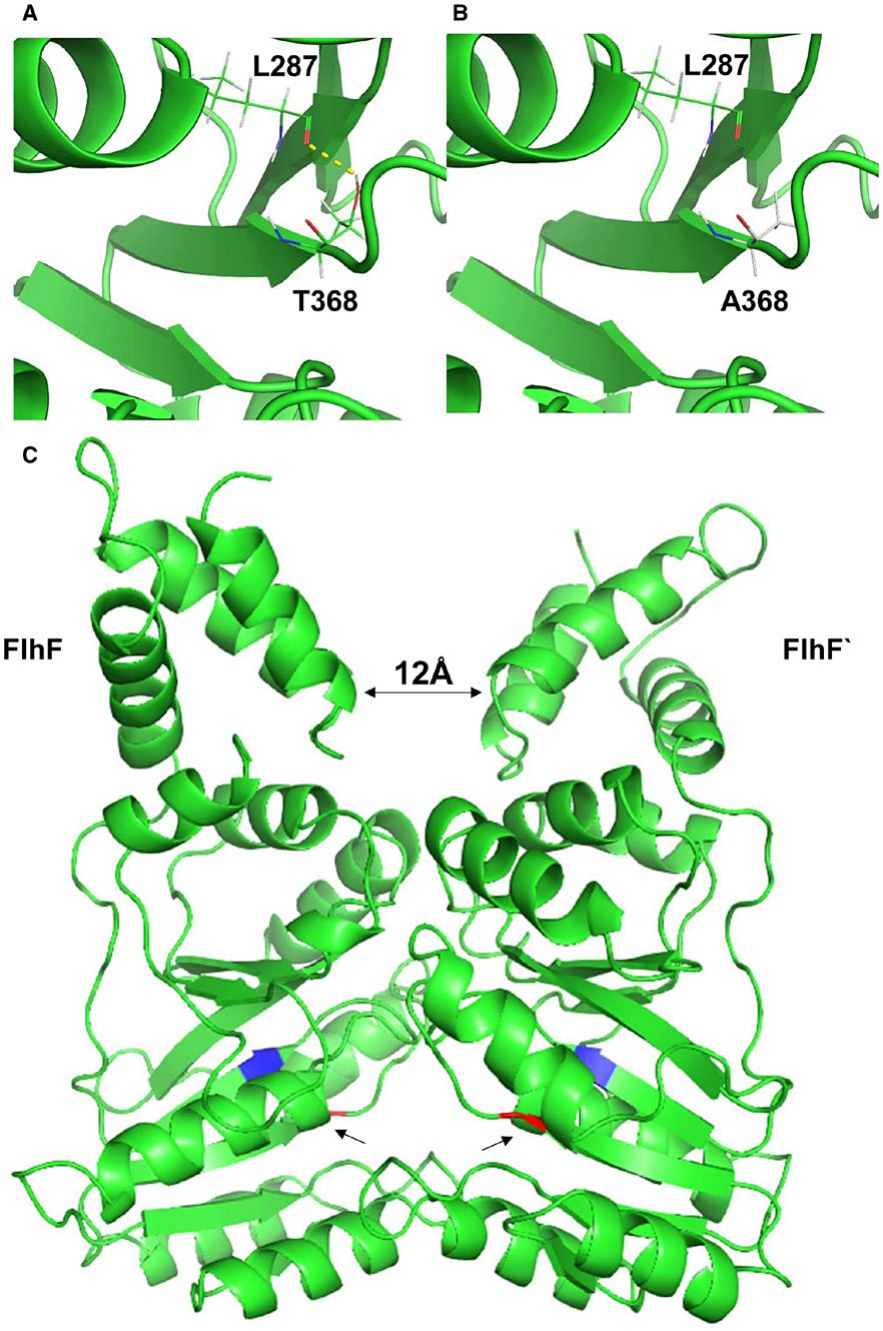
Predicted structures of FlhF and FlhF(T368A). Predicted location of the T368A mutation within a *C. jejuni *FlhF model derived from FlhF‐2px0.1.A using Swiss‐Model (Waterhouse *et al*., 2018) and rendered in PYMOL (DeLano, 2002). Panel A. Location of T368 in the FlhF wild type structure with the hydrogen bond marked as a yellow dotted line to the main chain oxygen of L287 (red). Panel B. Location of A368 in the FlhF(T368A) mutant protein that does not permit the formation of the hydrogen bond. Panel C. Predicted FlhF protein homodimer structure (FlhF and FlhF`) with the N domains orientated toward the top of the figure and the G domains at the bottom. The arrow heads in G domains indicate the locations of T368A substitution colored red in juxtaposition to L287 colored blue, to which the threonine residue is hydrogen bonded.

Bange *et al*. (2007) reported that FlhF of *B. subtilis* forms a stable homodimer structure in the presence of GTP, and that the G domains form a composite active site harboring two GTP molecules. Differences in the predicted protein structures of FlhF and FlhF(T368A) are presented, respectively, in Fig. 6A and B, which are based on models of the *C. jejuni* FlhF protein calculated from the template flagellar biosynthesis protein FlhF‐ 2px0.1 of *B. subtilis* (Bange *et al*., 2007). Substitution of the threonine residue with alanine prevents the formation of the hydrogen bond between L287 and T368 that tethers two adjacent β‐sheets that flank a returning loop that constitute the G3 domain. The effect of this is likely to destabilize GTP binding, and as a consequence the formation of the functional homodimer. Figure 6C shows the location of T368 at the monomer interface. The N domains remain free of the interface to enable interaction with other cellular components, and are separated by a distance of 12 Å in the dimer structure. The dependence of the G domains to stabilize the dimer is consistent with the reduced thermal stability and greater substrate dependence observed for the GTPase activity of FlhF(T368A). Impaired GTP binding would not only reduce the catalytic activity but the stability of any complex incorporating FlhF. Indeed wild type FlhF protein had a 2.3‐fold lower *K*_M_ for the GTP substrate than FlhF(T368A). The *k*_cat_ of 0.0115 s^–1^ for the FlhF protein was in excess of the *k*_cat_ of 0.007 s^–1 ^for FlhF(T368A).

The mechanism by which FlhF and FlhG regulate the polar flagella number and localization is not fully understood. In *V. cholera *FlhF was reported as solely required for the recruitment and correct localization of the flagellar MS ring inner membrane protein FliF, which is presumed one of the earliest flagellar structural components (Green *et al*., 2009). *C. jejuni* FlhF has also been proposed to operate at an early stage in flagellar biosynthesis on the basis that mutational changes that alter GTPase activity can lead to flagellar mislocalization or increased numbers of flagella (Balaban *et al*., 2009). The likely first step of flagellar biosynthesis is the formation of a multiple inner membrane protein complex, known as the flagellar export apparatus, which is able to secrete most of the flagellar proteins (Hendrixson, 2008), and it is to this complex that the GTP‐bound FlhF homodimer may be recruited to initiate flagellar biosynthesis. Once triggered, the flagellar export apparatus is proposed to release GDP‐bound FlhF, which can no longer act to stimulate flagellar formation (Schuhmacher *et al*., 2015). FlhF GTPase activity is stimulated by membrane located FlhG that itself is subject to ATP dependent homodimerization to affect flagellar assembly (Gulbronson *et al*., 2016). FlhF may also actively participate in the export assembly as a recent report demonstrates *flhF* deficient mutants unable to localize components of flagella export apparatus to the cell pole (Ren *et al*., 2018).

Surface structures are candidates for adhesion targets and receptors necessary for bacteriophage infection. In *C. jejuni,* flagella and capsular polysaccharide have been proposed to constitute essential attachment sites for the respective infection of group II and group III *Campylobacter* phages (Rakhuba *et al*., 2010; Sørensen *et al*., 2015). Group II phages CP220, Φ3 and Φ15 failed to infect non‐flagellated strains that included the largely non‐flagellated *flhF(T368A) *expressing strain. We further observed that group III phages showed a greater efficiency of plaque formation on wild type *C. jejuni *PT14 than the *flhF *inactivated mutant derivatives, suggesting that although flagella may not be essential, the presence of a functional flagella increases infection efficiency. Group III phages have been observed to enter the carrier state where the *flhF(T368A) *mutation would not strictly prevent phage infection but would certainly reduce the efficiency (Table 3).

Comparing the growth dynamics of group III phage infected wild type and *flhF* mutant *C. jejuni* PT14 in broth cultures demonstrated the mutant bacteria supported phage replication but did not exhibit reductions in the population count to the same degree as wild type. Complemetation of the *flhF* mutation with the *flhF *and *flhF(T368A) *allele restored wild type behavior with phage‐induced bacterial population crashes of 3.5 and 2.3 log_10_ CFU ml^–1^ respectively. The static period post lysis represents the growth stage when the probability of a bacterial cell division is similar to the chance of it being infected and lysed by phage particles. This period is reduced in the *flhF* mutant (2 h) compared to wild type (6 h), where the rate of infection is reduced. The static period was reinstated upon *flhF* complementation (6 h) but not so for *flhF(T368A)* complementation (4 h). The infection rate of the strain carrying *flhF(T368A)* is reduced. Calculation of the phage adsorption constants revealed a significant difference between the wild type and *flhF* mutant. The wild type complement of *flhF* regained the adsorption rate of the wild type, whereas the complement carrying *flhF(T368A)* remained similar to the inactivated *flhF* mutant. The root cause of these differences is the absence of the flagellar structure leading to inefficient interaction between the phage and the host bacteria.

Phage resistance and the formation of carrier state life cycle emerge under these conditions. The phase variable genes identified in the carrier state cultures (Table 1) are consistent with the emergence of phage resistance; *kpsC* mutants are impaired in capsular polysaccharide biosynthesis and have previously been reported to give rise to phage resistance (Coward *et al*., 2006), and the additional four phase variable genes identified have previously been reported to show shifts to phase‐off in bacteriophage escape mutants (Lis and Connerton, 2016). We have analyzed the frequency of carrier state formation in *C. jejuni *PT14 cells recovering post bacteriophage CP30A infection (the static phase). For wild type *C. jejuni *PT14 and the wild type complement PT14*flhF::kan 00230::flhF‐cat*, 7–10% of the recovered cells had entered carrier state life cycle on the basis of the continued association with phage CP30A. These carrier state cells showed resistance against super‐infecting phage CP220, Φ3, Φ15, CP30A, CPX, CLP6, and CLP47 having lost motility. However, no carrier state cells were recovered from the *flhF *mutant or the strain expressing *flhF(T368A). *These strains exhibit inefficient phage adsorption, which results in fewer infected cells compared with cells with intact flagella. These data suggest that the *flhF(T368A) *mutation does not provoke carrier state formation but is rather a consequence that enables limited motility in sub‐populations of cells that may be phage infected, while maintaining a reservoir of insensitive cells that will continue to divide in the presence of the phage. The acquisition of the *flhF(T368A) *mutation will also tend to maintain the specific phage association in the carrier state cultures as they appear to resist super‐infection. Phase variable genes that alter phage sensitivity will also act to prevent super‐infection and stabilize bacterial populations in the presence of high phage titers.

This study has demonstrated the independent selection of the point mutation *flhF(T368A) *in *C. jejuni *PT14CP30A carrier state isolates. FlhF(T368A) protein shows catalytically compromised GTPase activity with reduced stability that affects the ability of FlhF to produce a functional flagella. Non‐flagellated bacterial populations are resistant to group II bacteriophage and show diminished sensitivity to group III bacteriophage due to inefficient adsorption. However, *C. jejuni *carrying *flhF(T368A)* produce a subpopulation of bacteria with flagellar structures that can support phage adsorption and replication. Finely balanced structural/catalytic instability of the FlhF(T368A) GTPase represents a pivotal point in the cellular commitment to flagellar assembly that confers motility and phage sensitivity. The balance in carrier state cultures is tipped against motility in favor of phage insensitivity in the presence of high phage titers. Indeed CP30A carrier state bacteria are profoundly impaired in motility, and as a consequence exhibit poor cellular adhesion and invasion, and generally fail to colonize the intestinal tract of chickens (Hooton *et al*., 2016). However, at lower phage titers, as evident upon dilution in early exponential phase carrier state broth cultures, reversion to wild type motility may arise until the phage density increases and these motile bacteria become infected and lysed (Siringan *et al*., 2014). Under these circumstances reversion to the wild type allele will be subject to counter selection and the *flhF(T368A)* allele maintained. Consistent with this view we have used targeted amplicon sequencing to establish the dominance of the *flhF(T368A)* allele in carrier state isolates, and that the wild type allele can arise or persist but at low frequencies (≤ 10^–3^). The *flhF(T368A)* mutation is maintained because it produces adaptive phenotypes that allow bacteria and phage to coexist, and accord the abilities to escape phage super‐infection and survive extra‐intestinal environments (Siringan *et al*., 2014). We have demonstrated the complex mechanism by which carrier state cultures can arise and persist in *C. jejuni* due to the acquisition of a subtle mutation in a gene essential for the control of a key physiological attribute to this species; we expect that as other examples of bacteria/phage carrier state associations are established the nature of the mutational changes may vary but there will be parallels in the mechanisms that permit the host bacteria to express adaptive phenotypes to enable the bacteria and bacteriophage to coexist and survive.

## Experimental procedures

### Bacterial strains, growth conditions and antibiotic concentrations

*C. jejuni *PT14 and NCTC11168 were routinely grown on blood agar base No.2 plates (Oxoid, Basingstoke, UK) containing 5% (v/v) defibrinated horse blood (BA) at 42°C under microaerobic conditions (5% v/v oxygen, 2% v/v hydrogen, 88% v/v nitrogen, 5% v/v carbon dioxide) for 18 h. *Escherichia coli *TOP10, BL21DE3 Star and Origami 2 DE3 pLysS were cultured in Luria‐Bertani (LB) broth with appropriate antibiotic at 37°C with 150 r.p.m. shaking for 18 h. Kanamycin (25 μg ml^–1^) and chloramphenicol (12.5 μg ml^–1^) were used for selection of *C*. *jejuni*. For selection of *E. coli,* kanamycin (50 μg ml^–1^), chloramphenicol (25 μg ml^–1^) and ampicillin (100 μg ml^–1^) were applied as required.

### Whole genome and amplicon sequencing

Genomic DNAs from *C. jejuni *carrier state isolates PT14CP30ACS‐1, PT14CP30ACS‐2, and PT14CP30ACS‐3 recovered from biofilms and subsequently colony purified (Siringan *et al*., 2014), were prepared using a GenElute Bacterial Genomic DNA Kit (Sigma‐Aldrich, Gillingham, UK) from BA plate swabs. DNA sequencing was performed using the Illumina MiSeq platform. The data consisted of 2.2–2.8 million paired‐end sequence reads of 250 bp in length. Initial processing of the raw data, mapping of the sequence reads to *C. jejuni* PT14 (GenBank accession CP003871) and variant detection were performed using CLC Genomics Workbench version 8.0 (Qiagen, Aarhus, Denmark). The *flhF* gene sequence containing the *flhF(T368A)* allele was PCR amplified from each carrier state culture using *flhF(T368A)* primers (*flhF(T368A)* F, 5′‐CATGTTTGGTGTTTGCAG‐3′, and *flhF(T368A)* R, 5′‐AGAATCGGTGCAGTTGAG‐3′) in three independent reactions, which were pooled in equal volumes and purified using the Wizard SV Gel and PCR Clean‐Up System (Promega, Madison, USA). Sequence libraries were generated for paired‐end sequencing.

### Construction of C. jejuni PT14 flhF mutants

Wild type *flhF* and *flhF(T368A)* genes were PCR amplified from wild type *C. jejuni *PT14 and *C. jejuni *PT14CP30ACS respectively using *flhF* forward and reverse primers containing BsmBI and NgoMIV sites (*flhF* F, 5′‐CGTCTCACATGGTGATAAGTGGTGTGAGGTG‐3′, and *flhF* R, 5′‐GCCGGCTCATCTGGCACTTCTTGTCC‐3′). PCR products were cloned into pCR 2.1TOPO vector supplied from Invitrogen to create construct pCR2.1::*flhF* and pCR2.1::*flhF(T368A)*. Point mutation of *flhF(T368A)* was confirmed by PCR sequencing using *flhF*, *flhF(T368A)* and M13 universal primers, and aligning with the wild type *flhF* sequence.

To construct a *flhF* inactive mutant, a *flhF* cassette was first obtained by digesting pCR2.1::*flhF* with EcoRI. This cassette was then cloned as an EcoRI‐EcoRI fragment into an EcoRI‐digested pUC4K backbone to replace the kanamycin resistance gene to create pUC4KΔ*kan*::*flhF*. A kanamycin resistance cassette without a promoter was amplified from the pUC4k vector by PCR using primers containing XbaI sites (Kan F, 5′‐TCTAGAGCAAGGAACAGTGAATTGGAG‐3′, and Kan R, 5′‐TCTAGAGTGCGTAAGAACATAGAAAGG‐3′). The resulting kanamycin cassette was firstly cloned into pCR2.1TOPO, followed by digestion with XbaI to obtain the kanamycin resistance gene as a XbaI‐XbaI fragment and ligating the kanamycin resistance cassette into XbaI digested pUC4KΔ*kan*::*flhF* to construct pUC4KΔ*kan*::*flhF*::*kan*. This plasmid was naturally transformed into *C. jejuni *PT14 with the aim of targeting cross‐over of the *flhF *sequences flanking the kanamycin cassette to form *C. jejuni *PT14*flhF::kan*.

To create the *flhF *complementation constructs, BsmBI‐ NgoMIV fragments containing either *flhF* or *flhF(T368A) *were obtained by digesting pCR2.1::*flhF* or pCR2.1::*flhF*(T368A) with BsmBI and NgoMIV. The *flhF* and *flhF(T368A)* BsmBI‐ NgoMIV fragments were then inserted into BsmBI‐ NgoMIV digested pCfdxA::PerR plasmid (Gaskin *et al*., 2007) to form pCfdxA::*flhF* and pCfdxA::*flhF(T368A)* constructs. The constructs were naturally transformed into *C. jejuni *PT14*flhF::kan *and targeted to the pseudogene *Cj0046 (A911_00230) *using flanking homologous sequences within the vector.

### Natural transformation

Overnight grown *Campylobacter* was harvested into 10 ml Müller‐Hinton (MH) broth using sterile cotton swab and an OD_600_ reading of the suspension was measured. The bacterial concentration was then estimated based on optical density using an empirical equation previously stated by Scott (2006).

$$Campylobacter\;\mathrm{concentration}\left({\;\mathrm{CFUml}}^{-1}\right)\;=\;\left({\;\mathrm{OD}}_{600}\times \;2\;\times \;10^{9}\right)-\;6\;\times \;10^{6}Campylobacter\;\mathrm{concentration}\left({\;\mathrm{CFUml}}^{-1}\right)\;=\;\left({\;\mathrm{OD}}_{600}\times \;2\;\times \;10^{9}\right)-\;6\;\times \;10^{6}$$

Approximately 7.5 log_10_ CFU *Campylobacter *cells were dispensed in to the center of a MH blood agar plate and incubated at 42°C under microaerobic conditions for 6 h. A 1 μg aliquot of DNA were dispensed onto the surface of the bacteria halo and allowed to air dry. The plate was then incubated for another 18 h at 42°C under microaerobic conditions. After incubation for three to five days, the transformants were selected using MH blood agar with appropriate antibiotics, and further confirmed by polymerase chain reaction and DNA sequencing.

### Motility assay

Motility assay was carried out by stabbing an inoculum of overnight grown *C. jejuni* into the center of a semi‐solid motility agar plate (0.4% w/v MH agar) using a sterilized pipette tip. The plates were incubated at 42°C under microaerobic conditions for 48 h before the diameter of the motility halo was measured and recorded.

### Transmission electron microscopy

*C. jejuni* was firstly fixed with 3% (v/v) glutaraldehyde in 0.1 M cacodylate buffer, negative stained by uranyl acetate and then examined by TEM. To fix bacteria cells, fixative solution was prepared by adding 2.5 ml of 0.2 M cacodylate buffer, 1.9 ml of distilled water into 600 μl 25% (v/v) EM glutaraldehyde. Overnight grown *C. jejuni *cells were harvested into 600 μl of fixative solution using a 10 μl of inoculating loop. The *C. jejuni* pellet was fixed in the fixative solution at room temperature for 1 h and then centrifuged at 10,000 × *g* for one minute. The supernatant was discarded and 1 ml of 0.1 M cacodylate buffer was added to gently wash the pellet. The pellet was left in 0.1 M cacodylate buffer for 10 min and then centrifuged at 10,000 × *g* for one minute. The supernatant was removed and the pellet was then re‐suspended gently into 600 μl of 0.1 M cacodylate buffer. A 14 μl aliquot of the fixed *C. jejuni *suspension was transferred onto formvar/ carbon film on copper 200 mesh grid and left for 30 s. The suspension was removed by lens paper and 14 μl of 0.5% (w/v) uranyl acetate was added onto the grids to negative stain the *C. jejuni *cells for one minute. After staining, uranyl acetate was removed by lens paper and the grid was ready to be examined by TEM.

### Real‐time PCR

*C. jejuni *PT14, PT14*flhF::kan*, PT14*flhF::kan 00230*::*flhF‐cat* and PT14*flhF::kan 00230*::*flhF(T368A)‐cat* were grown on blood agar plates containing appropriate antibiotics at 42°C under microaerobic conditions for 18 h. Bacteria were then harvested into MH broth using a sterile cotton swab and the total RNA was isolated with Trizol reagent. First strand cDNA was subsequently synthesized by protocol modified from Untergasser (2008). Firstly, 20 μl of enzyme mix containing 8 μl of 5x First Strand Buffer, 4 μl of dithiothreitol (DTT), 2 μl of dNTPs (10 mM each), 1 μl of SUPERase inhibitor, 1 μl of SuperScript II and 4 μl of water was prepared and stored at room temperature. Annealing mix was prepared in 20 μl of volume containing 1 μg of total RNA, 25 ng of μl^–1^ random hexamers and the primers were annealed in a thermocycler at 70°C for 10 min followed by 25°C for 10 min. To which 20 μl of enzyme mix was then added to the reaction and the cDNA was synthesized in a thermocycler at 25°C for 10 min, 37°C for 45 min, 42°C for 45 min and 70°C for 15 min. The cDNA samples were diluted one in five prior to use in real‐time PCR.

To prepare samples for real‐time PCR, PowerUp SYBR Green Master Mix from ThermoFisher Scientific was used according to manufacturer’s protocol. Briefly, samples were prepared in a 96 well micro‐titer plate by mixing 10 μl of PowerUp SYBR Green Master Mix with 10 pmol of gene specific forward, reverse primer, and 2 μl of cDNA template in a total volume of 20 μl. Then real‐time PCR was carried out using a Light Cycler 480 instrument from Roche with settings of pre‐incubation at 95°C for 6 min; followed by 40 cycles of amplification including incubation at 95°C for 30 s, 58°C for 30 s and 72°C for one minute; and then one cycle of melting curve including incubation at 45°C for 5 s and 65°C for one minute; and the run was finished by cooling at 40°C for 30 s. The primer pairs used in real‐time PCR analysis were *flhF* F, 5′‐CCGTTGAAGATACAGAACAAAT‐3′, and *flhF* R, 5′‐GGCTACCATAACCTCATAAAG‐3′; *flaA* F, 5′‐CAGCTGAGTCACAAATCCGT‐3′, and *flaA* R, 5′‐CCATGGCATAAGAGCCACTT‐3′; *flaB* F, 5′‐GTTAAAGCAGCAGAATCAACCA‐3′, and *flaB* R, 5′‐ACTCATAGCATAAGAACCTGACTG‐3′; *flgD* F, 5′‐AATGGCTGGACAAGAAGTTCC‐3′, and *flgD* R, 5′‐CTCCATCGCTTGAACCACCA‐3′; *pflA* F, 5′‐TGCCTTATGTTGGAGCTTTGG‐3′, and *pflA* R, 5′‐TGTGCATCAATCACCACTTGA‐3′; and *flhG* F, 5′‐AGCGCGAATCTAGCCAATGT‐3′, *flhG* R, 5′‐AAGGAGCATTCTCCGCGTAA‐3′, *pgk *F, 5′‐TAGACGCATAAGATCAGCTATTCC‐3′ and *pgk *R, 5′‐ AAGTCTAGCAAGACGCTTAGC‐3′.

### Efficiency of plating

Bacteria were overnight grown on BA plates and subsequently harvested into 10 ml of 10 mM MgSO_4_ solution using a sterile cotton swab. From this suspension, 500 μl of aliquots were mixed with 5 ml of aliquots of NZCYM top agar tempered at 55°C and then poured onto a pre‐dried NZCYM plate. The plate was allowed to dry at room temperature and then incubated at 42°C for 30 min. After incubation, 10 μl of decimally diluted bacteriophage samples were spotted onto the bacterial lawn in triplicate and the spots were allowed to dry at room temperature. The plate was further incubated at 42°C under microaerobic conditions for 18 h. The plaques for each spot were measured and the plaque forming units per ml was then calculated.

### Enumeration of C. jejuni and bacteriophage

*C. jejuni* sample was ten‐fold serial diluted with MH broth and 10 μl of each dilution was spotted in triplicate onto a CCDA plate with 2% (w/v) agar to reduce swarming. After 48 h incubation at 42°C under microaerobic conditions, *C. jejuni *colonies were counted. To enumerate bacteriophage, the phage sample was ten‐fold serial diluted. A bacterial lawn of *C. jejuni *PT14 was prepared and 10 μl of each dilution phage sample was spotted onto the lawn in triplicate. Plates were then incubated at 42°C under microaerobic conditions for 48 h and plaques were counted.

### Growth characteristics of C. jejuni PT14 and its flhF mutants against bacteriophage CP30A

Overnight cultured *C. jejuni *PT14, PT14*flhF::kan*, PT14*flhF 00230::flhF‐cat* and PT14*flhF* *00230::flhF(T368A)‐cat* mutants were transferred into 100 ml of MH broth with appropriate antibiotics to give a final concentration of approximately 4 log_10_ CFU ml^–1^ and incubated at 42°C under microaerobic conditions with 150 r.p.m. shaking for 2 h. After incubation, bacteriophage CP30A was applied into each *C. jejuni* culture to give a final concentration of 10^2^ PFU ml^–1^ and the cultures were carried on incubating for another 24 h. Sample aliquots were removed every 2 h for *C. jejuni* and bacteriophage enumeration.

### One step growth curve for bacteriophage CP30A against C. jejuni PT14 and its flhF mutant derivatives

Overnight cultured *C. jejuni* PT14, PT14*flhF::kan*, PT14*flhF 00230::flhF‐cat* and PT14*flhF 00230::flhF(T368A)‐cat* mutants were transferred into 100 ml of MH broth with appropriate antibiotics to give a final concentration of approximately 7 log_10_ CFU ml^–1^ and incubated at 42°C under microaerobic conditions with 150 r.p.m. shaking for 2 h. The viable count was then measured after incubation as described above. For the experiment, bacteriophage CP30A was diluted and added to bacterial suspension at the titer of 10^6 ^PFU ml^–1^. The bacteria/ phage mix was further incubated at 42°C under microaerobic conditions with 150 r.p.m. shaking for 3 h and aliquoted samples were taken every 15 min. Aliquots were centrifuged at 13,000 × *g* for 5 min and the supernatant containing free phages were removed for enumeration. The adsorption constant was calculated using equation *k* = –ln (*P*_t_/*P*_0_)/*N*_t_, where *P*_t_ = phage titer at time point t (PFU ml^–1^), *P*_0_ = initial phage titer (PFU ml^–1^), *N* = bacterial viable count (CFU ml^–1^) and *t* = time (min).

### FlhF protein purification

Both *flhF* wild type and *flhF(T368A)* genes were PCR amplified including their promoter using *flhF* his_6_‐tag forward and reverse primer respectively containing in‐frame 5′ NdeI and BamHI site (*flhF *His‐tag F, 5′‐AACATATGGGACAACTTATACATACTT‐3′, and *flhF *His‐tag R, 5′‐AAGGATCCATTGCGAAGTTTATTTGCTTGG‐3′). The *flhF* PCR products were cloned into pCR 2.1 TOPO vector and the *flhF(T368A)* point mutation was confirmed by PCR sequencing. The *flhF* wild type and *flhF(T368A)* fragments were obtained by digesting the plasmids with NdeI‐BamHI and then ligated into a NdeI‐BamHI digested pET28b vector backbone to create N‐terminal His_6_‐tagged fusion. The pET28b construct containing wild type *flhF* was transformed into chemically competent *E. coli *BL21DE3 Star cells. The pET28b construct containing the His_6_‐tagged FlhF(T368A) gene was transformed into Origami2 DE3 pLysS cells to overcome inclusion body formation. To express the protein, a fresh colony harboring the pET28b*flhF* plasmid was cultured in 10 ml of LB broth at 37°C with 150 r.p.m. shaking for 18 h with the presence of kanamycin and chloramphenicol. After incubation, overnight grown culture was diluted 1:50 in one liter LB broth containing kanamycin, and chloramphenicol. The culture was incubated at 37°C with 150 r.p.m. shaking until OD_600_ reached 0.6 and then induced with isopropyl‐β‐D‐thiogalacto‐pyranoside (IPTG) at a final concentration of 0.4 mM at 37°C for 4 h. The bacteria cells were pelleted by centrifuging at 4,000 × *g* for 15 min at 4°C and then purified using a HisTrap HP column, supplied from GE Healthcare Life Sciences. Briefly, a pellet derived from one liter culture volume was resuspended in 10 ml of lysis buffer (50 mM Tris‐HCl, 150 mM NaCl, 20 mM imidazole, pH 7.2) containing 1 ml of lysozyme stock solution (10 mg ml^–1^) and 300 units of Benzonase Endonuclease supplied from Sigma‐Aldrich. The suspension was incubated on ice for 30 min and centrifuged at 12,000 × *g* for 30 min and 4°C. The clear lysate was collected and applied to HisTrap HP column, which was pre‐equilibrated with five column volumes of water and five column volumes of binding buffer (50 mM Tris‐HCl, 150 mM NaCl, 20 mM imidazole, pH 7.2). The column was then washed with 15 column volumes of binding buffer and eventually eluted with elution buffer (50 mM Tris‐HCl, 150 mM NaCl, 250 mM imidazole, pH 7.2) for five column volumes. After this, 500 μl of this protein was size fractionated into 24 fractions using Superose 12 chromatography (GE healthcare Life Sciences). Purified protein was analyzed by 12% sodium dodecyl sulfate‐polyacrylamide gel electrophoresis (SDS‐PAGE) against Precision Plus Protein Dual Color Standards (Bio‐rad) and protein concentration was measured by Bradford assay.

### GTP hydrolysis assay

The GTP hydrolysis assay was carried out using a High Throughput Colorimetric GTPase Assay Kit from Innova Biosciencs. Prior to the GTP hydrolysis assay, a standard curve was prepared to convert the absorbance reading into released *P*_i_ from hydrolysis reaction. A set of *P*_i_ standards was prepared to give a final concentration of 1, 2, 5, 7.5, 10, 20, 50 and 75 μM. Triplicate samples were prepared for each *P*_i_ concentration by mixing 200 μl of each standard with 50 μl of P_i_ColorLock in a 96‐well microtiter plate. After two minutes, 20 μl of stabilizer was added and mixed thoroughly. After 30 min, the absorbance at wavelength of 612 nm was measured and the standard curve of absorbance against *P*_i_ concentration constructed. For the GTP hydrolysis assay, the substrate buffer mix was first prepared in 100 μl of volume containing 100 mM Tris buffer, 5 mM MgCl_2_ and a fixed concentration of GTP. For the temperature assay, 0.25 mM of GTP was applied as a standard substrate concentration. The reactions were initiated by rapid mixing of 100 μl of enzyme with 100 μl of substrate buffer mix and incubated immediately at 4, 16, 25, 37, 42, 50, 60, 70 and 80°C for 30 min. A 50 μl of aliquot of P_i_ColorLock solution containing 0.5 μl of Accelerator solution was added to stop the enzyme reaction. After two minutes, 20 μl of stabilizer was added and mixed thoroughly to prevent color development from background *P*_i_. The color reached a maximum signal after 30 min incubation at room temperature and the absorbance of the sample was then determined at a wavelength of 612 nm in a Tecan plate reader. Pre‐heated samples were measured to determine the thermal stability of the enzyme activities. In this experiment, protein samples were heated at different temperatures for 5 min prior to mixing with substrate buffer. For substrate pre‐incubation 0.2 mM GTP was added to the incubation mixes at 42°C, and either stopped as a pre‐reaction control or diluted into the GTPase assay containing 0.5 mM GTP. The contribution of GTP self‐hydrolysis was monitored by control incubations without protein and the corrected enzymatic activities recorded. For kinetic assays, 0.05, 0.1, 0.25, 0.5 and 1 mM of GTP was used as substrate concentrations, and after 0, 2, 4, 6, 8 and 10 min the enzyme reaction at 42°C were stopped before the absorbance was measured. Released *P*_i_ was subsequently determined from OD_612_ readings against a standard curve. The elution buffer for the His_6_‐tag protein purification was applied as negative control in this enzymatic assay.

## Author contributions

LL carried out the experiments. LL and IC designed the study, interpreted the data and wrote the manuscript.
