# Supplementary material for: FlhF(T368A) modulates motility in the bacteriophage carrier state of Campylobacter jejuni
Source: Mol Microbiol. 2018 Oct 23;110(4):616–33. doi: 10.1111/mmi.14120 (PMC6282759; doi:10.1111/mmi.14120)
Supplement: Supplementary file 1 [file MMI-110-616-s001.pdf]

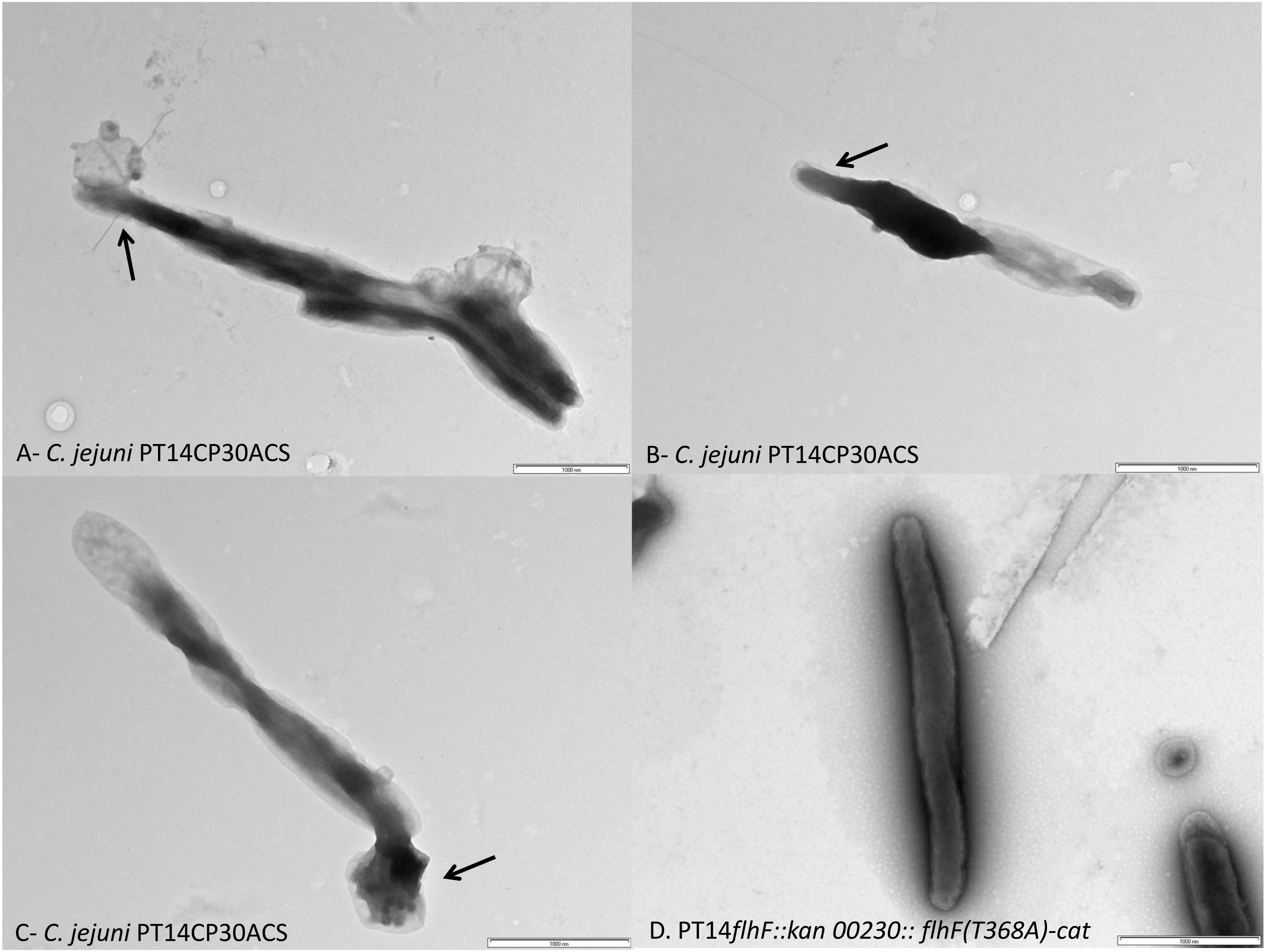

TEMs of C. jejuni. Panels A and B carrier state bacteria with arrows indicating a short non-polar flagellar; C carrier state with the arrow indicating encapsulated phage; D Typical non-flagellated flhF(T368) complement C. jejuni with reduced helical pitch.
